# Molecular dynamics approach to interfacial manipulation and dehydration enhancement of crude oil emulsions: a case study on plasma pre-treatment

**DOI:** 10.1039/d5ra06418j

**Published:** 2025-11-18

**Authors:** Chunhui Song, Wenjie Guo, Xuedong Gao, Qing Li, Huiru He, Minghe Chi

**Affiliations:** a Key Laboratory of Engineering Dielectrics and Its Application, Ministry of Education, Harbin University of Science and Technology Heilongjiang 150080 P. R. China chiminghe1985@hrbust.edu.cn; b PetroChina Company Limited Planning & Engineering Institute Beijing 100083 P. R. China

## Abstract

The current efficiency of crude oil dehydration is significantly influenced by the strength and stability of the oil–water interfacial film. To break through this bottleneck, this study first combined plasma pre-treatment with an electric field for the synergistic dehydration of crude oil emulsions. Experimental results indicated that plasma pre-treatment generated oxygen-containing derivatives, which significantly weakened the interfacial film, reducing interfacial tension by up to 43% and improving dehydration efficiency by 66.7%. Furthermore, combined with molecular dynamics simulations, we provided unprecedented mechanistic insights into the microscopic process. The simulations revealed that the oxygen-containing derivatives preferentially adsorb at the oil–water interface, reducing the electrostatic attraction and hydrogen bonding between water molecules inside the droplet, while simultaneously generating dispersion attraction with the oil phase. This dual action compromises the droplet's dynamic stability and facilitates severe deformation under the electric field. This study bridges a critical gap in the application of plasma technology to crude oil dehydration and establishes a theoretical framework for synergistic dehydration mechanisms involving multi-physical fields.

## Abstract 2

Plasma pre-treatment produces oxygen-containing derivatives that act at the oil-water interface to promote droplet deformation and coalescence, thereby enhancing electro-dehydration efficiency.

## Graphical abstract

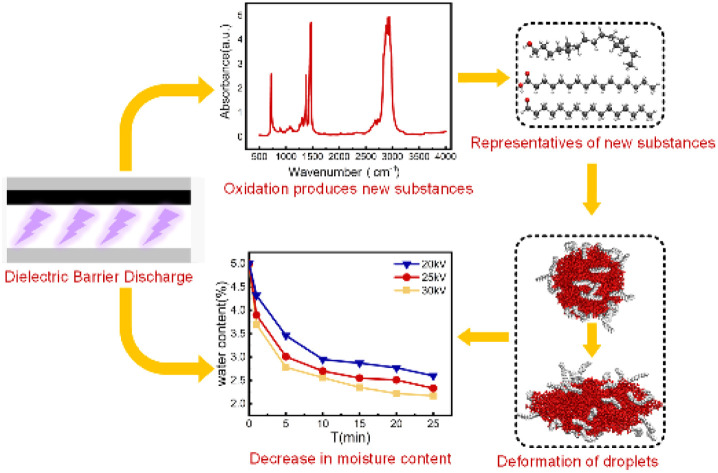

## 1. Introduction

Petroleum currently bears a critical role in the global energy landscape, making research on enhancing oil extraction efficiency of significant importance. As conventional oil field resources become increasingly depleted, the widespread application of enhanced oil recovery (EOR) technologies has improved recovery rates while simultaneously elevating the water cut of produced fluids.^1^ This has resulted in heightened stability of crude oil emulsions, significantly complicating oil–water separation processes.^2^ Electrocoalescence dehydration technology has emerged as a mainstream demulsification method due to its high efficiency and environmental friendliness, achieving separation through electric field-induced droplet deformation, coalescence, and sedimentation.^3^ However, the efficiency of this technique is constrained by the strength and stability of the oil–water interfacial film, where elevated interfacial tension and droplet dynamic stability severely inhibit the coalescence process.^4^ This limitation becomes particularly pronounced in emulsions following chemical flooding applications.^5^ Traditional pre-treatment methods, such as chemical demulsifiers, suffer from limitations including high energy consumption, secondary environmental contamination, and limited applicability,^6^ rendering them inadequate for targeted regulation of microscopic interfacial properties. Consequently, developing green and efficient novel interfacial modulation technologies to break through the separation bottleneck of highly stable emulsions has emerged as a core scientific challenge in petroleum extraction.

Plasma technology demonstrates significant potential in the energy and chemical engineering sectors due to its unique molecular activation capabilities.^7,8^ Characterized by high discharge efficiency, superior treatment homogeneity, precise process controllability, and environmental compatibility,^9^ its ionization processes generate abundant reactive species—including energetic electrons, ions, and free radicals—that effectively drive chemical reactions, leading to exploratory applications in liquid-phase processing.^10,11^ In the petroleum industry, established applications of plasma primarily focus on heavy oil upgrading and conversion, where the objective is to crack large hydrocarbon molecules to reduce viscosity and improve flowability, as demonstrated in the work of Prieto *et al.*^12,13^ and Shirazi *et al.*^14^ on plasma cracking.^15^ Another prominent application lies in the domain of water treatment and pollutant degradation, where plasma-generated reactive species produce potent oxidants that synergistically degrade organic contaminants.^16,17^

In contrast to these applications aimed at bulk chemical transformation or destructive degradation, the potential of plasma as a precision tool for interfacial manipulation in emulsions remains significantly underexplored. This gap is particularly critical given that chemical agents from enhanced oil recovery (EOR) operations strengthen oil–water interfacial films, thereby diminishing electrical dehydration efficiency and posing a major challenge for high water-cut oilfields.^18,19^ While plasma technology has demonstrated notable progress in areas like heavy oil upgrading,^20^ there is a paucity of mechanistic understanding regarding its use for targeted modulation of emulsion properties. This study, therefore, introduces a distinct application of plasma pre-treatment: not for bulk feedstock conversion, but as a green and efficient strategy to *in situ* generate interfacial-active, oxygen-containing derivatives that specifically weaken the oil–water interfacial film, thereby enhancing subsequent electro-coalescence dehydration. This approach addresses the core scientific challenge of breaking through the separation bottleneck of highly stable emulsions.

The introduction of plasma pre-treatment technology into crude oil electrical dehydration operates on the principle that high-energy particles targetedly weaken oil–water interfacial strength, thereby enhancing electric field coalescence efficiency.^21^ While this study addresses challenges involving discharge plasma characterization and analysis of interfacial physicochemical properties in oil–water emulsions, how plasma pre-treatment alters the microscopic dynamic behavior of emulsion droplets remains unrevealed. There exists a critical absence of molecular-level explanations for interfacial characteristics and droplet motion mechanisms, and the synergistic relationship between plasma pre-treatment and subsequent electrical dehydration remains unexplored. Consequently, this study proposes coupling plasma pre-treatment technology with electrical dehydration processes to achieve electrochemically enhanced demulsification. Through spectroscopic characterization and interfacial tension measurements, we elucidate the influence mechanisms of oxidative products derived from reactive species on emulsion stability. Simultaneously integrated with molecular dynamics simulations, this work analyzes microscopic characteristics—including intermolecular interaction energies and hydrogen bonding within emulsions—providing fundamental molecular-level insights into macroscopic phenomena. The research findings will advance the applicability of plasma technology in oil–water emulsion treatment and provide theoretical guidance for the development of electrical dehydration techniques under synergistic multi-physical fields, while establishing fundamental insights into their microscopic mechanisms. Additionally, an analytical system was integrated for comprehensive characterization of liquid-phase products. Fourier-transform infrared spectroscopy (FTIR) was utilized to identify oxygen-containing functional groups in the liquid products. Interfacial tension variations were quantified using a tensiometer *via* the pendant drop method. Water content dynamics in emulsions were monitored with a capacitance-based water-cut analyzer. Zeta potential measurements were conducted to assess interfacial stability through electrophoretic mobility analysis. Droplet size evolution was tracked using digital microscopy coupled with automated image processing.

## 2. Experimental and molecular simulation methods

### 2.1 Experimental section

#### 2.1.1 Construction of the experimental platform

**Fig. 1:**
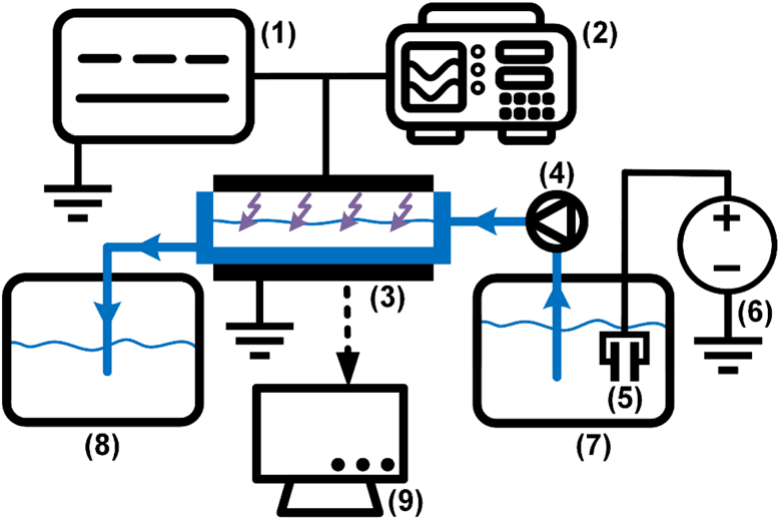
Schematic diagram of the experimental platform. (1) Industrial-frequency high-voltage power supply (model KZT10/0.22). (2) Oscilloscope (model TDS 3012B). (3) DBD electrode reactor. (4) Peristaltic pump (model YX-LP01-P100). (5) Electrical dehydration high-voltage source. (6) Planar dehydration electrodes. (7) Dehydration tank. (8) Liquid circulation cell. (9) Analytical instruments: FTIR spectrometer (model L1600400), tensiometer (model A101), zeta potential analyzer (model DT1202), water-cut analyzer (model KFO-30), digital microscope (model VHX-500FE).

The experimental platform was constructed to meet the research requirements for plasma pre-treatment and electrostatic dehydration of emulsions. It supports both dynamic and static plasma treatment modes, allowing direct observation of changes at the oil–water interface during plasma pre-treatment and the electro-dehydration process. Additionally, the system enables measurements of water content, interfacial tension, zeta potential, and droplet size distribution of the treated samples. To investigate the effects of plasma pre-treatment on the oil–water interface characteristics and the efficiency of electrostatic dehydration, an experimental platform was constructed as illustrated in Fig. 1. The core component of this setup is a single-layer dielectric barrier discharge (DBD) electrode reactor, which was custom-designed to meet the experimental requirements. This reactor generates plasma through gas-phase discharge under a strong electric field, enabling the treatment of crude oil *via* plasma. The main body of the reactor is made of quartz, allowing clear observation of the internal processes. The plasma is generated within the reaction chamber, where a stainless-steel high-voltage electrode plate is mounted on the upper section. The chamber contains the emulsion to be treated, with a maximum capacity of 40 mL of oil–water emulsion. The chamber contains the emulsion to be treated, separated from the high-voltage electrode by an air gap that can be adjusted freely. During the experiments, both the liquid level height and the air gap were consistently maintained at 4 mm. The quartz dielectric medium, with a thickness of 2.5 mm, features a smooth and burr-free surface to ensure a uniform electric field distribution. The high-voltage and ground electrodes, each having a diameter of 70 mm, are chamfered along the edges to prevent electric field concentration and avoid corona discharge, thereby reducing the risk of dielectric breakdown. The distance between the electrode plates and the crude oil can be freely adjusted *via* an electrode holder, allowing precise control of the air gap within the reaction chamber. The inlet and outlet ports of the reactor are connected to a peristaltic pump, which enables flexible regulation of the flow rate and velocity of the fluid, thereby facilitating the dynamic treatment of crude oil with plasma. The gas inlet and outlet ports on the upper cover are attached to an air pump that supplies air to the reaction chamber and simultaneously collects and treats exhaust gases to prevent environmental pollution.

#### 2.1.2 Experimental and detection equipment

A high-voltage power supply (KZT10/0.22) was used to apply a power-frequency AC voltage signal with an amplitude of 30 kV. For the dehydration process, vertically mounted plate electrodes were employed. The electrostatic dehydration high-voltage power supply was capable of outputting a DC voltage signal with an amplitude of 5 kV. A peristaltic pump was used to control the flow rate of the medium within a range of 0–1 L min^−1^. Additionally, a detection system was utilized for the analysis of liquid-phase products. An infrared spectrometer (L1600400) was applied to identify functional groups in the liquid-phase products. An interfacial tensiometer (A101) was used to measure changes in oil–water interfacial tension. A water content analyzer (KFO-30) was employed to monitor variations in the water content of the emulsion. A zeta potential analyzer (DT1202) was adopted to evaluate the interfacial stability of the emulsion. Finally, a digital microscope was used to observe changes in droplet size distribution.

#### 2.1.3 Experimental oils and reagents

To meet the experimental requirements, no. 10 industrial white oil was used as a model oil to investigate the oil–water interfacial properties. This industrial white oil consists primarily of C_16_–C_31_ normal and branched alkanes. It is colorless, odorless, chemically stable, and easy to observe. Its physical properties and chemical composition are similar to those of crude oil, making it a suitable analog for practical emulsions. The characteristics of the white oil are summarized in Table 1. To avoid interference from ions present in mineral water, distilled water was selected as the dispersed phase. Sorbitan monooleate (Span 80),^22^ a non-ionic oil-soluble surfactant with minimal influence on electric field distribution, was chosen to prepare emulsions with different water contents. Given that the industrial white oil is a complex mixture of C_16_–C_31_ alkanes, which complicates the analysis of plasma discharge characteristics, *n*-hexadecane was used instead to study the liquid-phase products under various discharge voltages and durations. The detailed parameters of all reagents are provided in Table 2.

**Table 1:** Physicochemical properties of industrial white oil

Dielectric constant (F m^−1^)	Density (m V^−1^)	Viscosity (Pa s^−1^)	Interfacial tension (mN m^−1^)
2.2	0.86	14	43

**Table 2:** Specifications of experimental reagents (AR grade: analytical reagent grade)

Reagent name	Specifications	Manufacturer
Industrial white oil	White oil no. 10	Morunke Lubricant Co., Ltd
*n*-Hexadecane	AR grade	Shanghai Macklin Biochemical Co., Ltd
Span 80	AR grade	Sinopharm Chemical Reagent Co., Ltd
*n*-Hexane	AR grade	Shanghai Macklin Biochemical Co., Ltd
Mineral oil	AR grade	Shanghai Macklin Biochemical Co., Ltd

#### 2.1.4 Calculation of discharge inception voltage

To determine the discharge inception voltage, the air within the gap was ionized into plasma acting on the gas–liquid interface once discharge occurred. The electrodes were connected to the high-voltage power supply *via* high-voltage leads, while a high-voltage probe linked to an oscilloscope enabled real-time monitoring of voltage, current, and discharge waveforms. The applied voltage at the onset of discharge in air was calculated using eqn (1) and (2) provided below.1
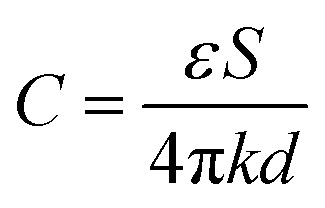
where *C* represents the capacitance, *ε* is the dielectric constant, *S* refers to the electrode area, *d* stands for the electrode distance, and *k* is Coulomb's constant.2
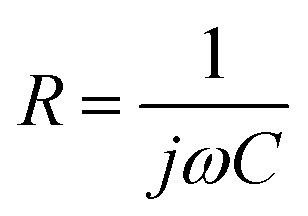
where *R* denotes the impedance, *j* is the imaginary unit, *ω* is the angular frequency, and *C* is the capacitance from eqn (1). The discharge inception voltage was estimated based on the critical electric field strength required for air ionization (3 kV mm^−1^) and the electrode gap distance (4 mm), yielding a theoretical breakdown voltage of approximately 12 kV. Considering field enhancement at electrode edges and other practical factors, the calculated applied voltage for discharge initiation was 15.36 kV, at which point the air began to ionize, generating plasma. During the experiment, when the RMS value of the power-frequency sinusoidal voltage approached approximately 15 kV, audible discharge sounds were observed along with faint discharge filaments, consistent with the theoretical prediction. Therefore, the experimental discharge inception voltage was set to an amplitude of 20 kV. Since surface discharge occurred when the applied voltage exceeded 35 kV, the operational voltage range for stable plasma generation was maintained between 20 kV and 30 kV.

#### 2.1.5 Experimental procedure

According to the required water content, water and white oil were sequentially added to a beaker, followed by the addition of 0.8 wt% Span 80. A high-speed shear machine (ESB-500) was used to emulsify the mixture at a speed of 9000 rad min^−1^ for 10 minutes to obtain a stable oil–water emulsion. The emulsion or white oil was then transferred to a circulation tank. The air pump and peristaltic pump were turned on to allow the emulsion to flow into the reaction chamber. Once the liquid level reached 4 mm, the voltage was applied. After the plasma treatment for the required duration, the sample was collected. The treated emulsion was then placed in an electrostatic dehydrator for dehydration, during which the water content was monitored in real time. Finally, experimental data were acquired using instruments such as an infrared spectrometer and a water content analyzer. The collected data were processed and analyzed in conjunction with experimental observations.

### 2.2 Molecular simulation

#### 2.2.1 Molecular model construction

**Fig. 2:**
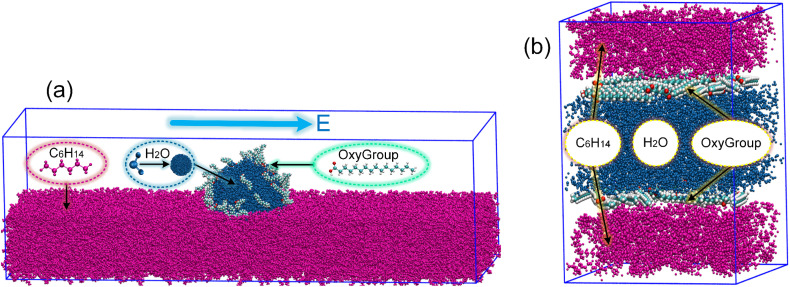
Initial molecular configurations of oil–water emulsions. (a) Single-droplet water-in-oil (W/O) model with the oil phase beyond *x* > 6 nm hidden for clarity. (b) Oil–water interfacial model.

It is important to note that different hydrocarbon phases were deliberately chosen for experiments and simulations to balance practical relevance with computational efficiency. In experiments, industrial white oil and *n*-hexadecane were used to represent the complexity of real crude oil and to facilitate liquid product analysis, respectively. For molecular dynamics simulations, *n*-hexane was selected as a computationally tractable model oil that captures the essential non-polar character of the hydrocarbon phase. This approach allows us to elucidate the fundamental mechanisms of oxygen-containing derivatives at oil–water interfaces while maintaining consistency with the experimental system through the use of C_16_-based OxyGroup molecules derived from *n*-hexadecane oxidation. To investigate the motion characteristics of emulsion droplets, following the study by Li *et al.*,^23^*n*-hexane (C_6_H_14_) was selected as a representative oil molecule in this work. The water droplet primarily consists of water molecules (H_2_O). A single water-in-oil emulsion droplet model was constructed as shown in Fig. 2a. A droplet with a diameter of 6 nm, containing 3760 water molecules, was placed at the center of a simulation box measuring 12 × 12 × 40 nm^3^. The droplet was surrounded by 25 000 *n*-hexane molecules as the oil phase. Additionally, 100 oxygen-containing derivative molecules carrying oxygen functional groups, hereafter collectively referred to as OxyGroup, were attached to the droplet surface. In the model, oxygen atoms are represented in red. Using OxyGroup as the sole variable, the influence of different oxygen-containing derivatives in the liquid products on the emulsion properties was analyzed by varying the types of OxyGroup. Molecules selected for the study included C_16_H_34_O (–OH), C_16_H_32_O (–CHO), and C_16_H_32_O_2_ (–COOH) as representative oxygen-containing derivatives. Five single-droplet systems with different OxyGroup types were constructed: pure, C6–OH, C6–CHO, C6–COOH, and mix. The pure system served as a control group with pure water and pure oil, while the mix system contained a mixture of all three oxygen-containing functional groups. A DC electric field of 0.7 V nm^−1^ was applied along the positive *z*-axis during the simulations. The specific method for applying the electric field was described in Section S1. Additionally, an oil–water interface model with dimensions of 6 × 6 × 8 nm^3^ was built to investigate interfacial characteristics, as shown in Fig. 2b. The model consisted of *n*-hexane oil phases on both sides, a water layer in the middle, and an interfacial layer of OxyGroup molecules at the oil–water boundary. The number of molecules was determined based on the number density from the single-droplet model. Similarly, five systems with varying OxyGroup types were established, following the same naming convention as the single-droplet systems.

#### 2.2.2 Molecular forcefield

Referring to previous similar MD studies, all simulations in this work were conducted using the GROMOS54A7 force field, which is well-recognized for its high accuracy in modeling oil–water emulsion systems.^24^ This force field employs the SPC/E water model,^25^ enabling an accurate description of key physical properties such as water density and diffusion constant. The intermolecular interactions within the system are primarily composed of electrostatic interactions (*E*^ele^_AB_) and van der Waals (vdW) interactions (*E*^vdW^_AB_), which are calculated using eqn (3) and (4), respectively.3
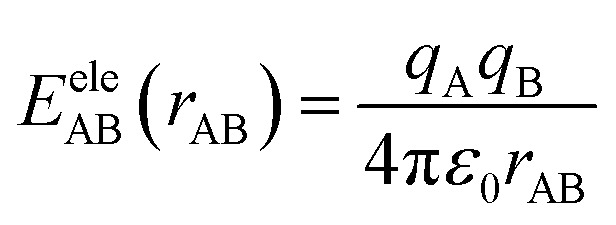
4
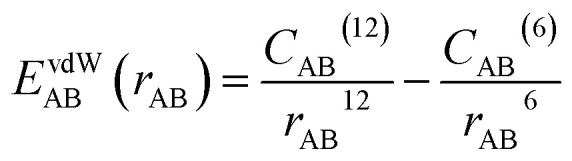
where *q*_A_ and *q*_B_ are the charges of atoms A and B, *C*_AB_^(12)^ and *C*_AB_^(6)^ are the Lennard-Jones (LJ) parameters between them, *r*_AB_ is the distance between them, and *ε*_0_ is the vacuum permittivity. The LJ parameters for a pair of atoms can be determined using eqn (5) and (6).5
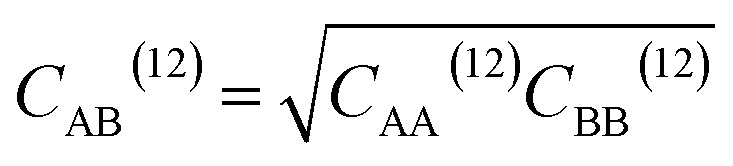
6
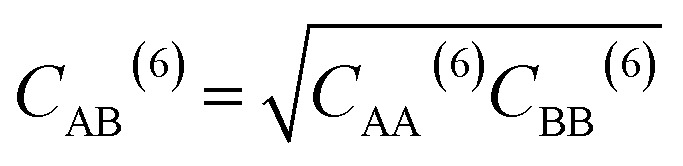
where *C*_AA_^(12)^ and *C*_AA_^(6)^ are the LJ parameters of atom A, *C*_BB_^(12)^ and *C*_BB_^(6)^ are the LJ parameters of atom B. For nonbonding parameters of all atoms, see Table S1.

#### 2.2.3 Simulation details

The molecular simulation workflow employed in this study is illustrated in Fig. S1. Energy minimization was first performed using the steepest descent algorithm to avoid simulation instability caused by excessively high repulsive forces within the system.^26,27^ As shown in Fig. S2 and Table S2, the potential energy of the system remained negative, and the maximum force acting on the atoms was below the specified threshold. Pre-equilibration was then conducted over 200 ps using the *V*-rescale and Berendsen methods to bring the system to the target temperature of 298.15 K and pressure of 100 kPa.^28,29^ Fig. S3–S5 demonstrate that both the temperature and pressure inside the simulation box stabilized around the set values, while the potential energy of the system also converged to a steady state. The production MD simulation was performed under the NPT ensemble using the *V*-rescale thermostat and the Parrinello–Rahman barostat for a duration of 1500 ps.^30,31^ The leap-frog integrator was employed with a time step of 1.0 fs. Electrostatic interactions were handled using the Particle Mesh Ewald (PME) algorithm to ensure computational accuracy.^32^ Periodic boundary conditions (PBC) were applied in all three dimensions to the system unit cell.^33^ The initial configuration was generated using the PACKMOL 18.169 package.^34^ All MD simulations were carried out with the GROMACS 2020.6 software,^35^ and the three-dimensional structural models were visualized using VMD 1.9.3.^36^

## 3. Results and discussion

### 3.1 Analysis of liquid phase products

**Fig. 3:**
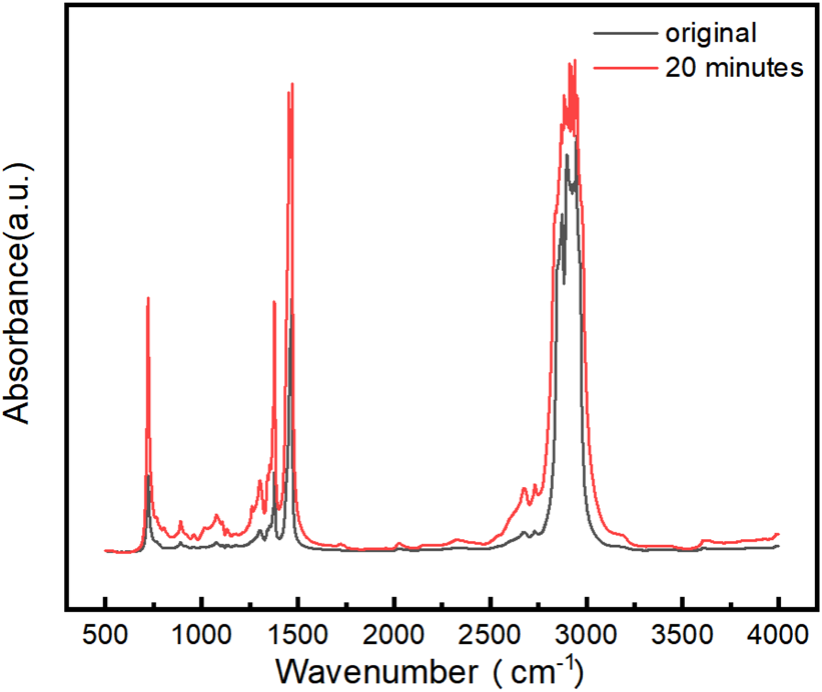
Evolution of FTIR spectra for liquid-phase products before and after 20 min plasma pre-treatment.

Composition analysis of liquid-phase products after plasma pre-treatment was conducted *via* Fourier-transform infrared spectroscopy,^37^ as presented in Fig. 3. The spectrum revealed emerging absorption bands in the 650–910 cm^−1^ region, indicative of aromatic compounds in the treated oil phase. A weak nascent peak at 1750 cm^−1^ signifies the formation of carbonyl functional groups (C=O). Broadened absorption between 2500–2800 cm^−1^ corresponds to oxygen-containing moieties, specifically carboxyl O–H stretching vibrations. Additionally, a faint band near 3600 cm^−1^ confirms the presence of hydroxyl groups (–OH). These findings confirm that oxidation reactions occurred in *n*-hexadecane following plasma pre-treatment. The underlying mechanism originates from chemically reactive species within discharge plasma—predominantly electronically excited oxygen molecules and energetic electrons—which exhibit strong oxidative capacity. This drives the progressive oxidation of alkanes, generating alcohols, aldehydes, carboxylic acids, and hydrocarbon radicals. This oxidative transformation constitutes the primary mechanism by which plasma alters the physicochemical properties of oil–water emulsions.

### 3.2 Analysis of emulsion dehydration efficiency

**Fig. 4:**
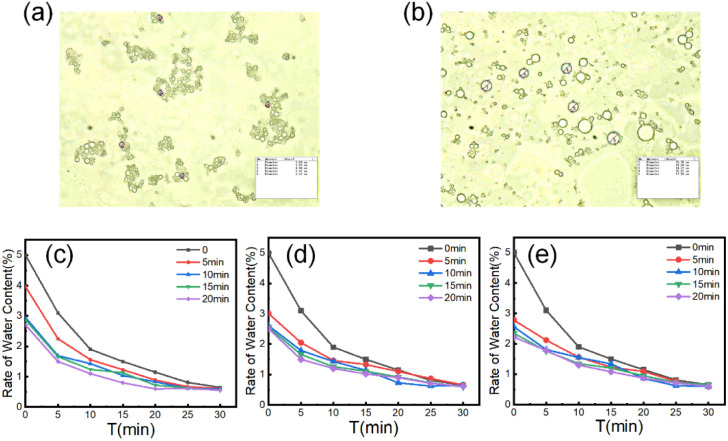
(a) Droplet size distribution pre-plasma treatment (median diameter ≈ 5 µm). (b) Droplet size distribution post-plasma treatment (median diameter ≈ 15 µm). (c–e) Dehydration performance at 2 kV cm^−1^ after plasma pre-treatment (initial water content = 5%). (c) Plasma discharge: 20 kV. (d) Plasma discharge: 25 kV. (e) Plasma discharge: 30 kV.

Variations in emulsion droplet size directly govern dehydration efficiency. Larger droplets exhibit enhanced deformation, coalescence, and gravitational sedimentation under electric fields, thereby improving dehydration performance.^38^ As depicted in Fig. 4a, droplet agglomeration occurred immediately without plasma pre-treatment. Individual droplets were indiscernible due to the formation of micro-droplet clusters, with diameters averaging approximately 5 µm. This phenomenon arises from elevated interfacial tension between droplets, which impedes coalescence during stochastic collision events—constituting the primary contributor to low electrical dehydration efficiency. Following plasma pre-treatment, as shown in Fig. 4b, droplet size remarkably increased to approximately 15 µm, with discrete individual droplets predominating. This significant coarsening demonstrates that the synergistic effect between plasma and electric fields facilitates effective droplet coalescence. As demonstrated in Fig. 4c–e, plasma pre-treatment significantly accelerates dehydration kinetics, with enhanced efficacy proportional to treatment duration. The most pronounced efficiency improvement occurs during the initial 10 minute phase, gradually attenuating as water content decreases. Critical data reveal that at 25 kV applied voltage, dehydration efficiency increased by 33.9%, 42.3%, 46.5%, and 66.7% for 5, 10, 15, and 20 minute pre-treatments respectively compared to untreated systems. This voltage level delivered optimal plasma-enhanced dehydration performance. Concurrently, reduced initial water content shortens requisite dehydration time, collectively enhancing overall emulsion separation efficiency.

### 3.3 Analysis of oil–water interfacial characteristics

**Fig. 5:**
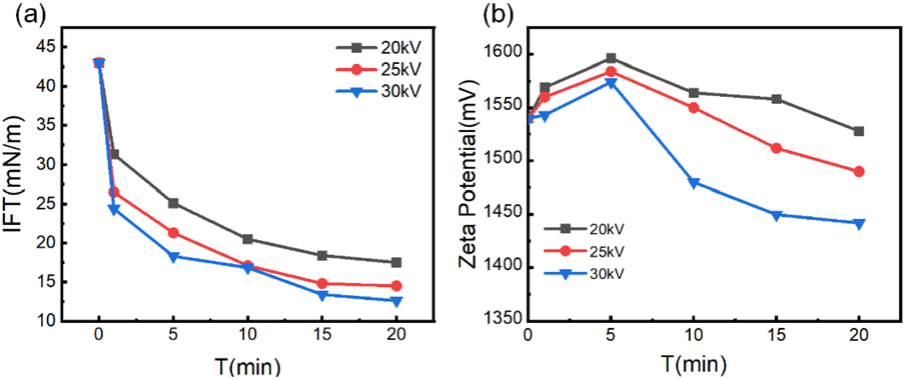
(a) Interfacial tension evolution of oil–water emulsions under varied discharge voltages (20–30 kV). (b) Zeta potential variation with discharge voltage (electrophoretic mobility analysis).

Interfacial tension serves as a critical parameter for characterizing emulsion stability and separation difficulty, representing the macroscopic manifestation of intermolecular interactions at oil–water interfaces.^39^ It exhibits a positive correlation with emulsion stability, while reduced interfacial tension facilitates water displacement. As shown in Fig. 5a, the interfacial tension of untreated emulsions measured 43 mN m^−1^. During the initial 10 minute pre-treatment phase, interfacial tension declined most rapidly—at 1 minute treatment under 30 kV, 25 kV, and 20 kV, reductions reached 43%, 38.4%, and 27.2% respectively. Beyond 10 minutes, the descent rate decelerated, stabilizing after 20 minutes. Higher applied voltages accelerated the reduction kinetics and yielded lower equilibrium interfacial tensions. This phenomenon originates from the cumulative effect of plasma discharge: prolonged treatment duration increases reactive collision frequency, intensifying chemical reactions that generate greater quantities of interfacial-active oxygenated derivatives. These derivatives migrate to oil–water interfaces, significantly reducing interfacial tension through modification of interfacial molecular interactions.

### 3.4 Analysis of emulsion stability

Zeta potential serves as a pivotal parameter for characterizing emulsion stability, reflecting the electrokinetic potential at oil–water interfaces. Its physical origin stems from the equilibrium between van der Waals forces and electrostatic interactions induced by surface charges on droplets, directly governing inter-droplet interaction strength.^40^ Investigating zeta potential evolution under plasma treatment is crucial for elucidating emulsion destabilization mechanisms.^41^ As shown in Fig. 5b, all curves under varying discharge voltages exhibit a transient rise in zeta potential within the initial 5 minutes of treatment, followed by a sustained decline approaching steady-state values after approximately 20 minutes. The initial rise phase indicates plasma-induced enhancement of interfacial molecular polarity, transforming the weakly polar oil–water interface into an oxygen-enriched polar interface. This transition originates from plasma-driven oxidation of alkanes, generating oxygen-containing functional groups. These groups adsorb at the interface to form an electric double layer (EDL), elevating surface charge density and causing transient zeta potential elevation. Subsequent potential decline correlates directly with droplet evolution. Continuous action of oxygenated groups promotes coalescence of small droplets, leading to significant size enlargement. Larger droplets possess diminished specific surface area, reducing adsorbed charge density per unit area and consequently lowering the measured zeta potential.

### 3.5 Microscopic mechanisms of emulsion droplet dynamics

**Fig. 6:**
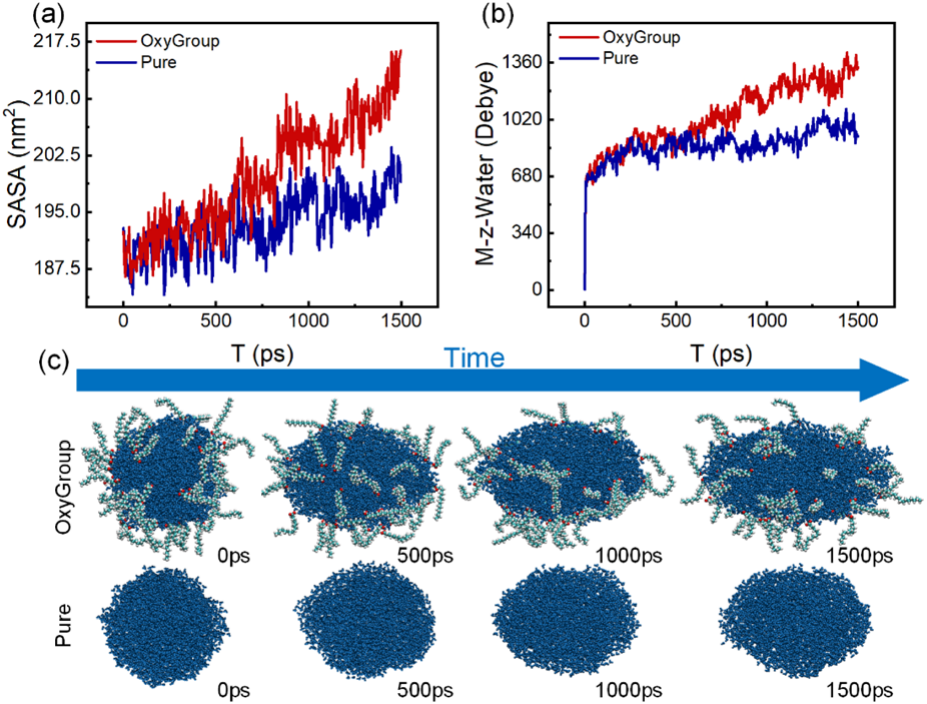
(a) Evolution of solvent accessible surface area (SASA) of emulsion droplets under OxyGroup effects. (b) Variation in total dipole moment along the *z*-axis induced by OxyGroup interactions. (c) Comparative VMD visualization of droplet deformation. Top: OxyGroup system. Bottom: Pure system (time interval: 500 ps; total simulation: 1500 ps).

The solvent-accessible surface area (SASA) provides an approximate metric for quantifying temporal evolution of nanodroplet surface area.^42^ For specific details on SASA, see Section S4. Integrated with VMD-visualized deformation trajectories, SASA offers direct insight into droplet distortion extent. As depicted in Fig. 6a, OxyGroup-modified droplets exhibit progressive SASA expansion over time, indicating continuous axial stretching. This phenomenon is further corroborated by the evolution of the droplet's dipole moment along the electric field direction. Fig. 6b reveals that intense water molecule polarity induces instantaneous dipole realignment under electric fields, triggering an abrupt surge and sustained growth in molecular dipole moments. Concurrently, enhanced intermolecular repulsive forces drive water migration within droplets, amplifying deformation. As demonstrated in Fig. 6c, OxyGroup-modified droplets exhibit substantially greater elongation along the electric field direction compared to the pure system, confirming that stretching directly induces surface area expansion. Crucially, hydrophilic functional groups project toward the aqueous core while hydrophobic alkyl chains extend into the oil phase, revealing the interfacial adsorption mechanism of OxyGroup at oil–water interfaces. This configuration critically enhances droplet deformation and promotes electro-coalescence, providing microscopic validation of the macroscopically observed significant droplet enlargement in Section 3.2.

**Fig. 7:**
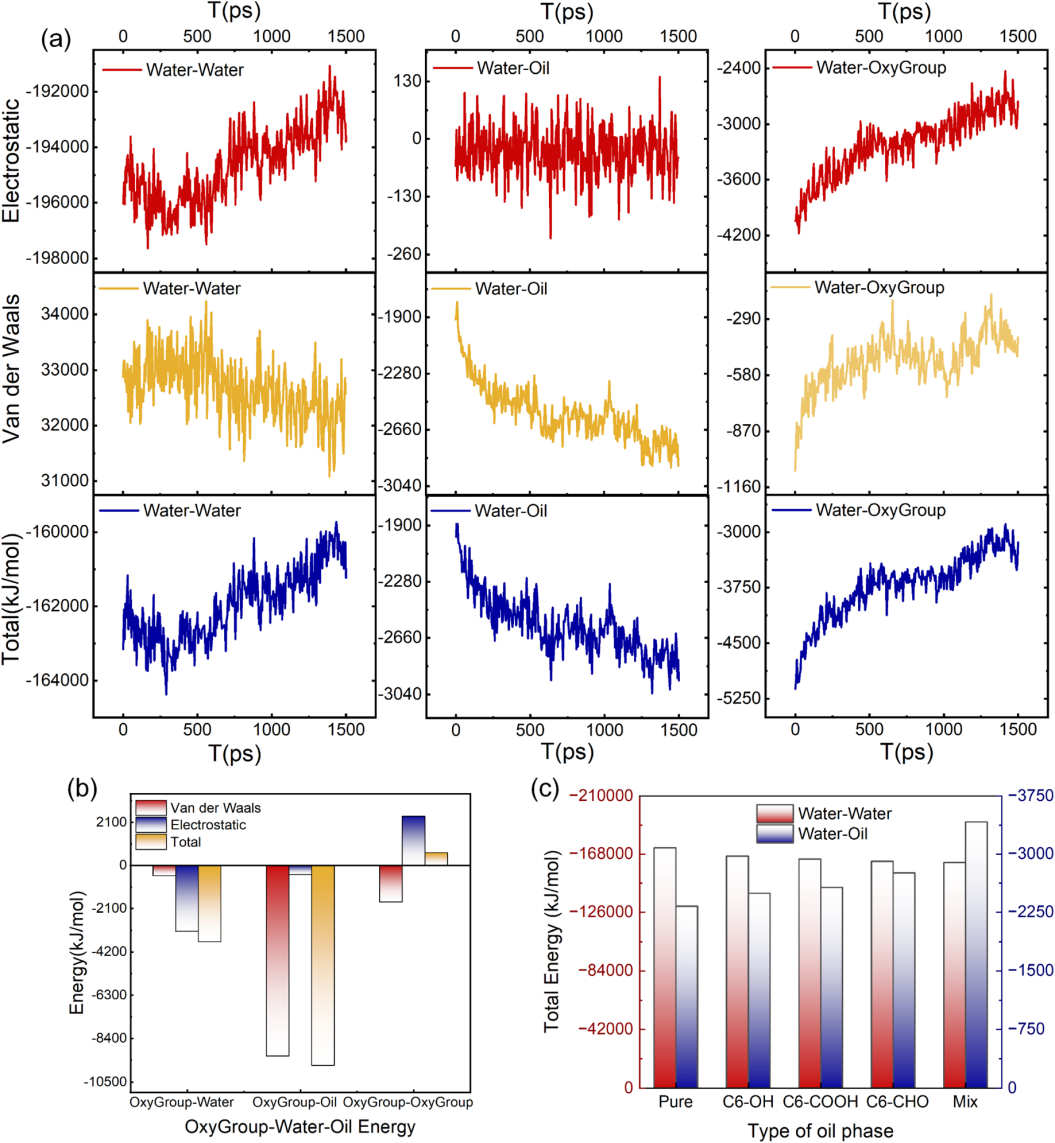
(a) Interaction energy components pertaining to the emulsion droplet. (b) OxyGroup-specific interaction energy contributions. (c) Comparative interaction energies across distinct oil-phase systems.

Droplet dynamics exhibit strong dependence on intermolecular interaction energies.^43^ Analysis of these energies within the model provides deeper mechanistic insights into deformation processes. As shown in Fig. 7a, both electrostatic attraction and exchange repulsion progressively weaken within OxyGroup-modified droplets over time. This reduction diminishes cohesive constraints among water molecules, thereby facilitating droplet stretching—a conclusion corroborated by deformation morphology in Fig. 6c. Critical observation of Fig. 6c further reveals that during intensified deformation, a fraction of OxyGroup molecules migrates into the oil phase away from the droplet interface, consequently weakening their interactions with the aqueous core. As evidenced in Fig. 7a, electrostatic attraction between OxyGroup and water molecules diminishes during OxyGroup migration away from the droplet interface. Concurrently, dispersion attraction between the oil phase and droplet progressively intensifies. This energy redistribution promotes water migration toward the oil phase, further amplifying droplet deformation. Fig. 7b reveals that the interaction energy between *n*-hexane molecules and OxyGroup is dominated by dispersion attraction. Compared to water molecules, *n*-hexane exhibits stronger affinity for OxyGroup, driving its migration into the oil phase. During this process, OxyGroup entrains adjacent water molecules, augmenting deformation. Among deformation-influencing factors, reduced electrostatic attraction within the droplet constitutes the primary contributor, while enhanced oil-droplet dispersion attraction acts as a secondary factor. As revealed in Fig. 7c, the pure system exhibits the highest electrostatic interaction energy among water molecules, indicating maximal electrostatic constraints. This constitutes the primary reason for the resistance to deformation and coalescence in oil–water emulsion droplets. The attenuating effect on electrostatic interactions varies among OxyGroup types, with the C6–CHO system demonstrating the second strongest electrostatic screening after the mix system. Critically, the mix system shows the lowest intermolecular electrostatic energy, signifying the weakest cohesive constraints between water molecules and consequently the most pronounced droplet deformation. This molecular-level evidence directly accounts for the reduced zeta potential and transient emulsion instability observed in Section 3.4.

**Fig. 8:**
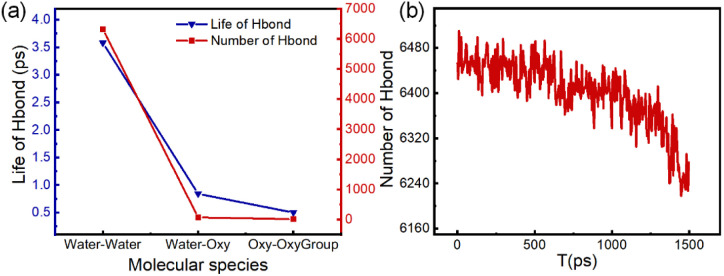
Hydrogen bonding network characteristics. (a) Intermolecular hydrogen bonding in C6–COOH system. Number and lifetime of H-bonds. Types: water–water, water–OxyGroup (water–Oxy), OxyGroup–OxyGroup (Oxy–OxyGroup). (b) Changes in the number of hydrogen bonds between water molecules in droplets.

Due to the presence of hydroxyl groups (–OH) in water molecules and oxygen-containing functional groups (*e.g.*, –OH, –CHO, –COOH) in OxyGroup species, these moieties facilitate hydrogen bonding between distinct molecules or within individual molecules.^44^ Taking the C6–COOH system as an exemplar, its carboxylic acid group (–COOH) comprises both carbonyl (C=O) and hydroxyl (–OH) subunits. The highly electronegative oxygen atoms in the carbonyl group possess lone electron pairs, enabling them to serve as hydrogen bond acceptors, while the hydroxyl group acts as a hydrogen bond donor. This dual functionality promotes robust hydrogen bonding with water molecules. As illustrated in Fig. 8a, hydrogen bonding interactions exist both among water molecules and within OxyGroup species. The positively charged hydrogen atoms of water molecules form hydrogen bonds with the highly electronegative oxygen atoms in the –OH groups of adjacent water molecules. These bonds exhibit a lifetime of 3.58 ps, contributing significantly to maintaining the spherical stability of droplets. Concurrently, hydrogen bonds form between H/O atoms of water molecules and O/H atoms of OxyGroup moieties, with a lifetime of 0.84 ps. This indicates robust adsorption of OxyGroup at the droplet interface, constituting a key mechanism for its action at the oil–water interface. Additionally, intermolecular hydrogen bonding within OxyGroup species (lifetime: 0.50 ps) facilitates the formation of a mechanically coherent interfacial film on the droplet surface. Hydrogen bond lifetimes reflect the dynamic equilibrium between bond rupture and reformation, which directly governs droplet stability. As evidenced in Fig. 8b, the population of water–water hydrogen bonds progressively declines under OxyGroup influence. This reduction indicates that during droplet deformation, increased intermolecular distances between water molecules deteriorate hydrogen bonding conditions, thereby weakening cohesive forces among water molecules. Consequently, the droplet's capacity to maintain structural integrity diminishes, promoting deformation. These findings demonstrate that OxyGroup compromises internal droplet stability by reducing both hydrogen bond density and lifetime, validating the observed phenomenon of intensified droplet deformation.

### 3.6 Microscopic mechanisms of oil–water emulsion interfacial characteristics

**Fig. 9:**
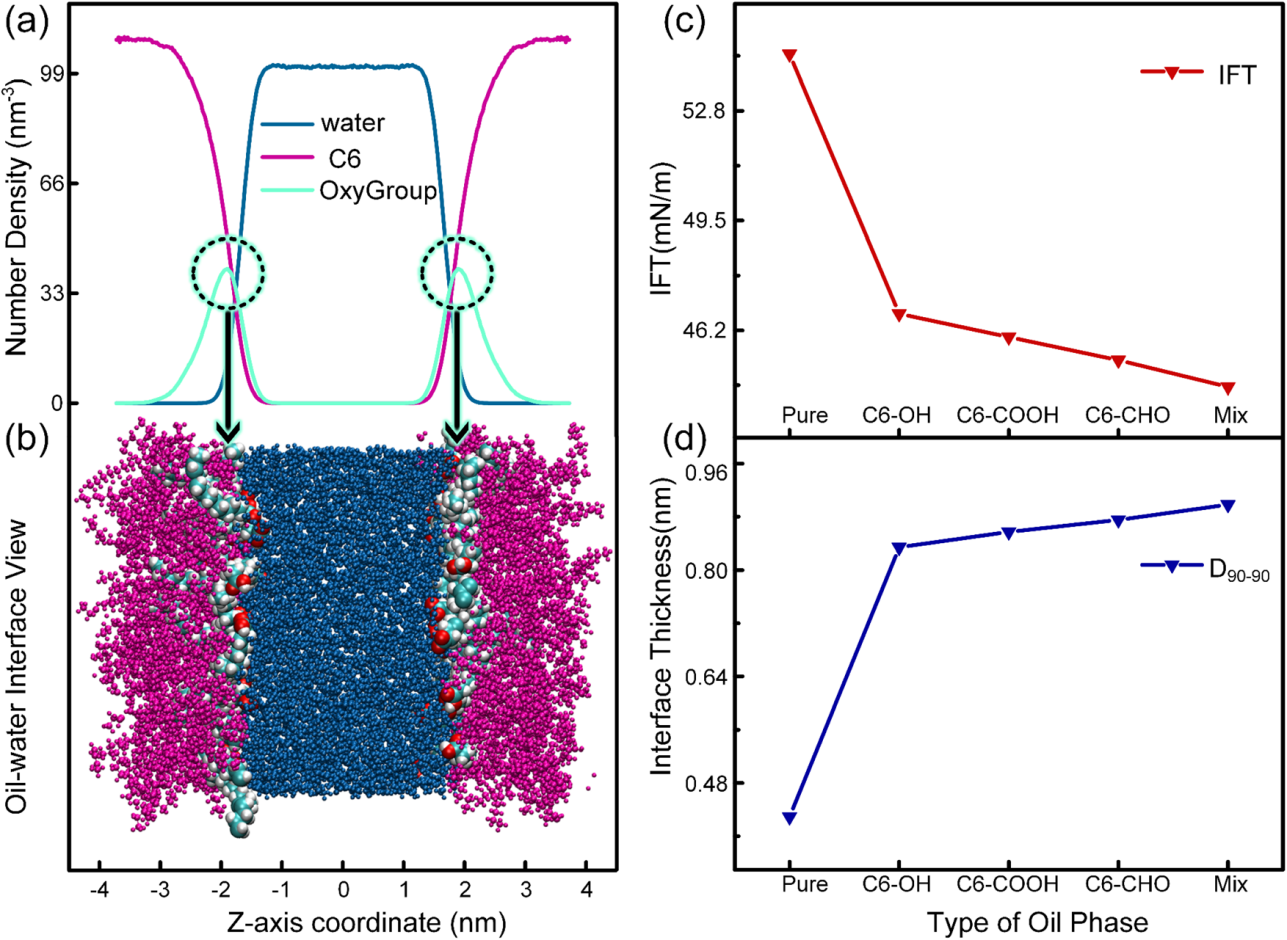
(a) Molecular number density profiles at oil–water interface under OxyGroup effects. (b) VMD visualization of interfacial configuration with OxyGroup adsorption. (c) Interfacial tension across simulated systems. (d) Interfacial thickness comparison of different systems.

Given the challenges in extracting droplet surface pressure, an oil–water interfacial model (Fig. 2b) was employed to approximate interfacial tension calculations for emulsified droplets. Simulation results in Fig. 9b reveal enhanced homogeneity in the mixture of OxyGroup species at the oil–water interface. Distinctively, hydrophilic functional groups extend into the aqueous phase while hydrophobic alkyl chains penetrate the oil phase. This surfactant-like spatial distribution enables directional alignment at the interface, forming a transitional molecular interphase layer. Molecular number density distributions across the interfacial region were analyzed to elucidate this spatial arrangement, as shown in Fig. 9a. A distinct overlap between aqueous and oleic phases was observed, with the OxyGroup-derived interfacial film localized within the oil–water transition zone. This phenomenon indicates that OxyGroup promotes partial mutual solubilization of water and oil molecules, which attenuates interfacial repulsion forces and consequently reduces interfacial tension. The interfacial tension was calculated using eqn (7) given below.^45^7
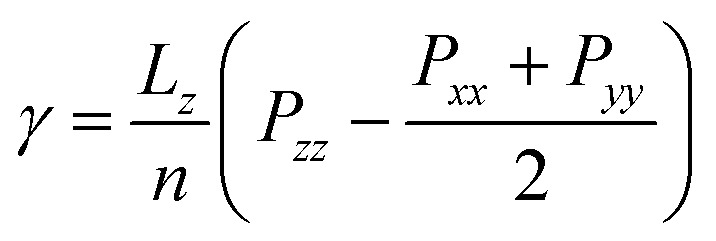
where *L*_*z*_ is the dimension of the system in the *z* direction, *n* is the number of interfaces, and *P*_*zz*_, *P*_*xx*_ and *P*_*yy*_ are the pressure components in the *z*, *x* and *y* directions, respectively. Interfacial tension serves as a macroscopic manifestation of imbalanced molecular forces at biphasic interfaces, directly governing droplet morphological stability. In Fig. 9c, the pure system exhibits the highest oil–water interfacial tension (54.5 mN m^−1^), whereas systems containing OxyGroup show significant reductions. Notably, the C6–CHO system demonstrates a tension of 45.3 mN m^−1^—slightly higher than the mix system but substantially lower than pure—demonstrating that OxyGroup modulates interfacial characteristics under electric fields by reorganizing molecular arrangements and interactions, effectively reducing interfacial tension. Interfacial thickness was derived from molecular number density profiles using the “90–90” criterion—defined as the distance spanning from 90% oil phase concentration to 90% aqueous phase concentration. As depicted in Fig. 9d, the incorporation of OxyGroup species significantly increased interfacial thickness across all systems. The mix system exhibited the most pronounced increase (0.898 nm), followed by C6–CHO (0.875 nm), while the pure system showed minimal thickness (0.429 nm). This demonstrates that OxyGroup modifies interfacial structural properties through dual-interface adsorption.

### 3.7 Comparative perspective: plasma pre-treatment *vs.* chemical additive injection

A pertinent question arising from this study is whether the observed enhancement in dehydration efficiency could be achieved by the direct injection of oxygen-containing additives, circumventing the need for plasma pre-treatment. While both approaches aim to modify the oil–water interface, the plasma-assisted strategy offers distinct and synergistic advantages that transcend mere chemical addition. Plasma operates as an *in situ* and on-demand generator of a complex mixture of interfacial-active species. Our FTIR analysis and molecular simulations confirm the formation of diverse oxygenated derivatives, including alcohols, aldehydes, and carboxylic acids. Such a mixture likely exhibits synergistic interfacial activity due to the varying hydrophilic–lipophilic balance values of its components, potentially leading to more robust and efficient interfacial coverage compared to single-component surfactants or simple mixtures.^46,47^ This complex composition is difficult to replicate by simple additive injection.

However, the plasma pre-treatment process offers distinct and synergistic advantages that may extend beyond the action of single-component additives. Our molecular dynamics simulations revealed that the mix system, incorporating a combination of different OxyGroup molecules, resulted in the most significant reduction in interfacial tension and the weakest cohesive forces within water droplets compared to systems with a single type of OxyGroup. This suggests that the complex mixture of various oxygenated derivatives generated *in situ* by plasma may exhibit superior performance due to synergistic effects at the interface, potentially achieving a more comprehensive disruption of the stable emulsion film than any single chemical agent.

Furthermore, the plasma approach is inherently a green and reagent-free process. Unlike chemical demulsifiers, which require synthesis, transportation, and precise dosing—potentially leading to secondary pollution—the plasma technique generates active species directly from the feed gas and the oil itself through electrical discharge. This aligns with the growing demand for sustainable and environmentally friendly technologies in the petroleum industry.^48^ Therefore, while the addition of specific oxygenated compounds might replicate certain aspects of interfacial tension reduction, the plasma pre-treatment represents a more integrated and potentially more effective strategy by combining *in situ* generation of a multi-component active agent with the benefits of a purely physical treatment process.

## 4. Conclusions

In this work, we have systematically combined macroscopic experiments with atomistic molecular dynamics simulations to investigate the efficacy and mechanism of dielectric barrier discharge (DBD) plasma pre-treatment for enhancing the electro-dehydration of water-in-crude oil emulsions. This multi-scale approach allowed us to correlate quantitative macroscopic efficacy with underlying molecular interactions, thereby providing a more comprehensive understanding from observable phenomena to atomistic-level events. The key findings can be summarized as follows:

Our experimental results collectively demonstrate that plasma pre-treatment effectively weakens the rigid oil–water interfacial film. The oxidation of hydrocarbon components by high-energy reactive species appears to generate interfacial-active oxygen-containing derivatives *in situ*. This chemical modification led to a significant reduction in interfacial tension (by up to 43% after 1 min at 30 kV) and a marked improvement in electro-coalescence efficiency, where dehydrated water droplets grew to approximately 15 µm and the dehydration efficiency improved by up to 66.7% compared to untreated systems. These data suggest that plasma pre-treatment is a promising modality for enhancing separation.

The molecular dynamics simulations provided crucial mechanistic insights that help to bridge the gap between macroscopic observation and molecular-level action. The simulations suggest a dual-action mechanism: the hydrophobic moieties of the plasma-induced derivatives (OxyGroup) generate substantial dispersion attraction with the oil phase, likely driving droplet migration. Simultaneously, their presence at the interface was observed to reduce the electrostatic attraction and disrupt the hydrogen bonding network among water molecules within the droplet. This combination of reduced internal cohesion and enhanced external pull is consistent with the observed compromise in the droplet's dynamic stability and its facilitated deformation under an electric field, thereby explaining the enhanced coalescence.

A key factor in the interfacial tension reduction is inferred to be the spontaneous, oriented arrangement of OxyGroup molecules at the oil–water interface, where their hydrophobic alkyl chains extend into the oil phase while the electronegative oxygen-containing groups face the aqueous phase. This surfactant-like configuration forms a transitional molecular interphase layer, which likely promotes partial mutual solubilization and attenuates interfacial repulsive forces, as supported by the measured increase in interfacial thickness.

This study helps to bridge a research gap in the application of plasma technology to crude oil electrical dehydration. The synergistic framework explored here, combining plasma pre-treatment with electric fields, along with the mechanistic understanding provided by the multi-scale methodology, offers valuable insights for the development of future high-efficiency dehydration techniques. The findings are expected to inform the rational design of plasma-based processes for complex fluid separation in the petroleum and related industries.

## Conflicts of interest

There are no conflicts to declare.

## Supplementary Material

RA-015-D5RA06418J-s001

## Data Availability

The data supporting this article have been included as part of the supplementary information (SI). Supplementary information: the SI file contains detailed methodological descriptions for the molecular dynamics simulations performed in this study, including: the application method of the DC electric field; full nonbonded force field parameters for Span-80, n-hexane, and water molecules; a step-by-step description of the simulation process (energy minimization, pre-equilibration, and production runs) with supporting figures and tables demonstrating system convergence; and the definition and calculation method for the solvent accessible surface area (SASA). See DOI: https://doi.org/10.1039/d5ra06418j.

## References

[cit1] Olabode O., Alaigba D., Oramabo D., Bamigboye O. (2020). Modelling Low-Salinity Water Flooding as a Tertiary Oil Recovery Technique. Model. Simulat. Eng..

[cit2] Ahmadi P., Asaadian H., Kord S., Khadivi A. (2019). Investigation of the simultaneous chemicals influences to promote oil-in-water emulsions stability during enhanced oil recovery applications. J. Mol. Liq..

[cit3] Li M., Yang D., Lv S., Zhao X., Wang J., Xia M., He L. (2024). Understanding the enhancement mechanism of critical electric field strength for salt-laden droplets electric coalescence through synchronous coupled magnetic field. Colloids Surf., A.

[cit4] Zheng L., Cao C., Chen Z., Cao L., Huang Q., Song B. (2020). Evaluation of emulsion stability by monitoring the interaction between droplets. LWT--Food Sci. Technol..

[cit5] Liu J., Liu S., Zhong L., Yuan S., Wang Q., Wei C. (2023). Study on the emulsification characteristics of heavy oil during chemical flooding. Phys. Fluids.

[cit6] Qin J., Wei C., Wei T., Li Z., Pang Z., Luo P., Feng C., Qiu G., Wei C., Wu H., Peng Y., Jiang C., Preis S. (2022). Evolution of biochemical processes in coking wastewater treatment: a combined evaluation of material and energy efficiencies and secondary pollution. Sci. Total Environ..

[cit7] Joshi G., Mir A. Q., Layek A., Ali A., Aziz S. T., Khatua S., Dutta A. (2022). Plasmon-Based Small-Molecule Activation: A New Dawn in the Field of Solar-Driven Chemical Transformation. ACS Catal..

[cit8] Kaushik T., Ghosh S., Dolkar T., Biswas R., Dutta A. (2024). Noble Metal Plasmon-Molecular Catalyst Hybrids for Renewable Energy Relevant Small Molecule Activation. ACS Nanosci. Au.

[cit9] Ambujakshan A. T., Pringle J. M., Chen Z., Corr C. S., Plessis J. d., Hodgson P. D., Dai X. J. (2018). An environmentally friendly in situ plasma and anodization method to produce titanium dioxide nanotubes. Plasma Processes Polym..

[cit10] Hamans R. F., Parente M., Baldi A. (2021). Super-Resolution Mapping of a Chemical Reaction Driven by Plasmonic Near-Fields. Nano Lett..

[cit11] Stefancu A., Gargiulo J., Laufersky G., Auguié B., Chiş V., Le Ru E. C., Liu M., Leopold N., Cortés E. (2023). Interface-Dependent Selectivity in Plasmon-Driven Chemical Reactions. ACS Nano.

[cit12] Khatibi M., Nahil M. A., Williams P. T. (2024). Interaction of furfural and hexadecane as bio-oil and plastics pyro-oil model compounds with non-thermal plasma processing as a route to in-situ hydrogen donor upgrading of bio-oil. Biomass Bioenergy.

[cit13] Prieto G., Okumoto M., Shimano K., Takashima K., Katsura S., Mizuno A. (2001). Reforming of heavy oil using nonthermal plasma. IEEE Trans. Ind. Appl..

[cit14] Taghvaei H., Shirazi M. M., Hooshmand N., Rahimpour M. R., Jahanmiri A. (2012). Experimental investigation of hydrogen production through heavy naphtha cracking in pulsed DBD reactor. Appl. Energy.

[cit15] Wang L., Yang Y., Sun J., Xin Y., Zhu X., Sun B. (2023). Liquid phase plasma for in-situ hydrogenation of heavy oil model compound (n-hexadecane). J. Anal. Appl. Pyrolysis.

[cit16] Lee J. W., Ji Y. Y., Sohn D. K., Ko H. S. (2021). Enhancement of continuous bubbles by non-thermal plasma for water treatment. J. Mech. Sci. Technol..

[cit17] Attri P., Tochikubo F., Park J. H., Choi E. H., Koga K., Shiratani M. (2018). Impact of Gamma rays and DBD plasma treatments on wastewater treatment. Sci. Rep..

[cit18] Nguyen D., Sadeghi N., Houston C. (2012). Chemical Interactions and Demulsifier Characteristics for Enhanced Oil Recovery Applications. Energy Fuels.

[cit19] Jia D., Zhang J., Sun Y., Wang S., Gao S., Qiao M., Li Y., Qu R. (2023). Collaboration between Oil Development and Water/Power Consumption in High-Water-Cut Oilfields. Sustainability.

[cit20] Gimžauskaitė D., Tamošiūnas A., Aikas M., Uscila R. (2023). Thermal plasma potential to remediate soil contaminated with diesel. Environ. Res..

[cit21] Khosravi R., Rodriguez C., Mostowfi F., Sieben V. (2020). Evaluation of crude oil asphaltene deposition inhibitors by surface plasmon resonance. Fuel.

[cit22] Koneva A. S., Safonova E. A., Kondrakhina P. S., Vovk M. A., Lezov A. A., Chernyshev Yu. S., Smirnova N. A. (2017). Effect of water content on structural and phase behavior of water-in-oil (n-decane) microemulsion system stabilized by mixed nonionic surfactants SPAN 80/TWEEN 80. Colloids Surf., A.

[cit23] Li N., Sun Z., Pang Y., Qi Z., Liu W., Li W., Sun M., Li B., Wang Z. (2022). Microscopic mechanism for electrocoalescence of water droplets in water-in-oil emulsions containing surfactant: a molecular dynamics study. Sep. Purif. Technol..

[cit24] Humphrey W., Dalke A., Schulten K. (1996). VMD: visual molecular dynamics. J. Mol. Graph..

[cit25] Schmid N., Eichenberger A. P., Choutko A., Riniker S., Winger M., Mark A. E., van Gunsteren W. F. (2011). Definition and testing of the GROMOS force-field versions 54A7 and 54B7. Eur. Biophys. J..

[cit26] Exl L., Bance S., Reichel F., Schrefl T., Stimming H. P., Mauser N. J. (2014). LaBonte's method revisited: an effective steepest descent method for micromagnetic energy minimization. J. Appl. Phys..

[cit27] Furuya A., Fujisaki J., Shimizu K., Uehara Y., Ataka T., Tanaka T., Oshima H. (2015). Semi-Implicit Steepest Descent Method for Energy Minimization and Its Application to Micromagnetic Simulation of Permanent Magnets. IEEE Trans. Magn..

[cit28] Bussi G., Donadio D., Parrinello M. (2007). Canonical sampling through velocity rescaling. J. Chem. Phys..

[cit29] Eslami H., Mojahedi F., Moghadasi J. (2010). Molecular dynamics simulation with weak coupling to heat and material baths. J. Chem. Phys..

[cit30] Huang C. C., Chatterji A., Sutmann G., Gompper G., Winkler R. G. (2010). Cell-level canonical sampling by velocity scaling for multiparticle collision dynamics simulations. J. Comput. Phys..

[cit31] Seliya P., Bonn M., Grechko M., Experimental M. (2023). Access to Mode-Specific Coupling between Quantum Molecular Vibrations and Classical Bath Modes. J. Phys. Chem. Lett..

[cit32] Zhou R., Harder E., Xu H., Berne B. J. (2001). Efficient multiple time step method for use with Ewald and particle mesh Ewald for large biomolecular systems. J. Chem. Phys..

[cit33] Kostritskii A. Y., Alleva C., Cönen S., Machtens J.-P. (2021). g_elpot: A Tool for Quantifying Biomolecular Electrostatics from Molecular Dynamics Trajectories. J. Chem. Theory Comput..

[cit34] Martínez L., Andrade R., Birgin E. G., Martínez J. M. (2009). PACKMOL: a package for building initial configurations for molecular dynamics simulations. J. Comput. Chem..

[cit35] Van der Spoel D., Lindahl E., Hess B., Groenhof G., Mark A. E., Berendsen H. J. C. (2005). GROMACS: fast, flexible, and free. J. Comput. Chem..

[cit36] Meyer N., Piquet V., Wax J.-F., Xu H., Millot C. (2019). Rotational and translational dynamics of the SPC/E water model. J. Mol. Liq..

[cit37] Andersen S. I., Mahavadi S. C., Abdallah W., Buiting J. J. (2017). Infrared Spectroscopic Analysis of the Composition of an Oil/Water Interfacial Film. Energy Fuels.

[cit38] Chakraborty M., Bart H.-J. (2006). Emulsion liquid membranes: role of internal droplet size distribution on toluene/n-heptane separation. Colloids Surf., A.

[cit39] Song F., Zheng H., Li T., Fu X., Feng C., Ma C., Jiang S., Wang J., Huang Y., Zhou F. (2024). The influence of asphaltene and resin on the stability of crude oil emulsion and its demulsification mechanism. J. Mol. Liq..

[cit40] Yang Z., Jiang C., Xiang Q., Wu J., Li J., Cao Z., Xiao F. (2025). Probing the stability of emulsified asphalts: a dual analysis of zeta potential and particle size. Fuel.

[cit41] Li D., Wang T., Chen S., Liu Q., Xie Y., Liu C. (2020). Experimental Investigation on Droplet Deformation and Breakup under Uniform DC Electric Field. Microgravity Sci. Technol..

[cit42] Eisenhaber F., Lijnzaad P., Argos P., Sander C., Scharf M. (1995). The double cubic lattice method: efficient approaches to numerical integration of surface area and volume and to dot surface contouring of molecular assemblies. J. Comput. Chem..

[cit43] Li B., Guo Z., Li N., Wang D., Li G., Zheng L., Qi B., Jiao T. (2023). Molecular dynamics simulation of wax deposition in crude oil systems. Colloids Surf., A.

[cit44] Li B., Guo Z., Li N., Wang D., Li G., Zheng L., Qi B., Jiao T. (2023). Mode and mechanism of water droplet breakup in oil under high-voltage and high-frequency pulsed electric fields. J. Mol. Liq..

[cit45] Mikami Y., Liang Y., Matsuoka T., Boek E. S. (2013). Molecular Dynamics Simulations of Asphaltenes at the Oil–Water Interface: From Nanoaggregation to Thin-Film Formation. Energy Fuels.

[cit46] Akram W., Zhang X., Wang Z., Mostafa M. Y. M., Gunawardane A., Yang Z., He L., Sui H. (2025). Understanding the demulsification of highly waxy shale oil emulsions by oxygen-rich demulsifiers. Fuel.

[cit47] Ma J., Li X., Zhang X., Sui H., He L., Wang S. (2020). A novel oxygen-containing demulsifier for efficient breaking of water-in-oil emulsions. Chem. Eng. J..

[cit48] Adewunmi A. A., Nazar M., Mahmoud M., Patil S., Hussain S. M. S., Kamal M. S. (2025). Advanced ionic liquid as a sustainable demulsifier for crude oil emulsions. Energy Fuels.

