# Supplementary material for: Molecular dynamics approach to interfacial manipulation and dehydration enhancement of crude oil emulsions: a case study on plasma pre-treatment
Source: RSC Adv. 2025 Nov 18;15(53):45014–25. doi: 10.1039/d5ra06418j (PMC12624851; doi:10.1039/d5ra06418j)
Supplement: RA-015-D5RA06418J-s001 [file RA-015-D5RA06418J-s001.pdf]

Supplementary Information (SI) for RSC Advances.
This journal is © The Royal Society of Chemistry 2025

Molecular dynamics approach to interfacial manipulation and dehydration enhancement of crude oil emulsions: a case study on plasma pre-treatment

Chunhui Song [a], *Wenjie Guo* [a], *Xuedong Gao* [b], *Qing Li* [b], *Huiru He* [a], *Minghe Chi* [a,*]

[a] Key Laboratory of Engineering Dielectrics and Its Application, Ministry of Education, Harbin University of Science and Technology, Heilongjiang, China, 150080, P. R. China

[b] PetroChina Company Limited Planning & Engineering Institute, Beijing, China, 100083, P. R. China

[*] Corresponding author. E-mail: chiminghe1985@hrbust.edu.cn

S1. DC electric field application method

The electric field utilized in this study was generated by the built-in program within GROMACS software. The mathematical expression for this specific electric field is depicted in Eq. (S1).[1]

$$E(t) = E_0 \exp\left[-\frac{(t - t_{a0})^2}{2\sigma_p{}^2} \right] \cos\left[\omega_p (t - t_{a0}) \right] \tag{S1}$$

where E_0 is the amplitude of electric field strength, t_{a0} is the time at the peak in the field strength, t is electric field application time, σ_p is the width of the pulse, and ω_p is the angular frequency. When σ_p=0 and ω_p=0, a DC electric field with a field strength of E_0 will be generated. The simulation system employed DC electric fields exclusively along the z-axis of the box.

S2. Force field parameters

The force field parameters for Span-80, n-hexane and water molecules were shown in Table S1.[2,3]

Table S1 Nonbonded interaction parameters.

$C_{24}H_{44}O_6$			
Atom	C_6 (kJ mol^{-1} nm^6)	C_{12} (kJ mol^{-1} nm^{12})	Charge (e)
H44	8.464e-05	1.5129e-08	0.0481
C24	0.0023406244	4.937284e-06	-0.2379
H42	8.464e-05	1.5129e-08	0.0481
H43	8.464e-05	1.5129e-08	0.0481

C22	0.0023406244	4.937284e-06	0.2314
H38	8.464e-05	1.5129e-08	-0.0473
H39	8.464e-05	1.5129e-08	-0.0472
C19	0.0023406244	4.937284e-06	-0.0623
H34	8.464e-05	1.5129e-08	0.0015
H35	8.464e-05	1.5129e-08	0.0015
C16	0.0023406244	4.937284e-06	0.111
H28	8.464e-05	1.5129e-08	-0.0227
H29	8.464e-05	1.5129e-08	-0.0227
C13	0.0023406244	4.937284e-06	-0.1758
H24	8.464e-05	1.5129e-08	0.0491
H25	8.464e-05	1.5129e-08	0.0491
C14	0.0023406244	4.937284e-06	0.0445
H26	8.464e-05	1.5129e-08	0.0031
H27	8.464e-05	1.5129e-08	0.0031
C17	0.0023406244	4.937284e-06	-0.1579
H30	8.464e-05	1.5129e-08	0.0332
H31	8.464e-05	1.5129e-08	0.0332
C20	0.0023406244	4.937284e-06	0.2933
H36	8.464e-05	1.5129e-08	-0.0251
H37	8.464e-05	1.5129e-08	-0.0251
C23	0.0023406244	4.937284e-06	-0.3374

H41	8.464e-05	1.5129e-08	0.1479
C21	0.0023406244	4.937284e-06	-0.251
H40	8.464e-05	1.5129e-08	0.1186
C18	0.0023406244	4.937284e-06	0.1888
H32	8.464e-05	1.5129e-08	0.0059
H33	8.464e-05	1.5129e-08	0.0059
C12	0.0023406244	4.937284e-06	-0.2044
H22	8.464e-05	1.5129e-08	0.0481
H23	8.464e-05	1.5129e-08	0.0481
C10	0.0023406244	4.937284e-06	0.0641
H18	8.464e-05	1.5129e-08	0.0069
H19	8.464e-05	1.5129e-08	0.0069
C8	0.0023406244	4.937284e-06	-0.023
H14	8.464e-05	1.5129e-08	0.0086
H15	8.464e-05	1.5129e-08	0.0086
C7	0.0023406244	4.937284e-06	0.0091
H12	8.464e-05	1.5129e-08	0.0065
H13	8.464e-05	1.5129e-08	0.0065
C9	0.0023406244	4.937284e-06	0.0217
H16	8.464e-05	1.5129e-08	0.0324
H17	8.464e-05	1.5129e-08	0.0324
C11	0.0023406244	4.937284e-06	-0.4016

H20	8.464e-05	1.5129e-08	0.1367
H21	8.464e-05	1.5129e-08	0.1367
C15	0.002025	1e-06	0.7662
O6	0.00308914	4.77422e-06	-0.6026
O5	0.0022619536	1.21e-06	-0.4477
C6	0.002025	1e-06	0.1274
H7	8.464e-05	1.5129e-08	0.0711
H8	8.464e-05	1.5129e-08	0.0711
C4	0.0023406244	4.937284e-06	0.1106
H4	8.464e-05	1.5129e-08	0.1263
O4	0.00177494	1.21e-06	-0.6876
H11	0	0	0.4591
C1	0.0023406244	4.937284e-06	0.2188
H1	8.464e-05	1.5129e-08	0.0185
O1	0.0022619536	1.21e-06	-0.4594
C5	0.0023406244	4.937284e-06	0.0073
H5	8.464e-05	1.5129e-08	0.0802
H6	8.464e-05	1.5129e-08	0.0802
C3	0.0023406244	4.937284e-06	0.3395
H3	8.464e-05	1.5129e-08	-0.009
O3	0.00177494	1.21e-06	-0.7365
H10	0	0	0.445

C2	0.0023406244	4.937284e-06	0.283
H2	8.464e-05	1.5129e-08	0.0387
O2	0.00177494	1.21e-06	-0.7035
H9	0	0	0.406

C_6H_{14}

Atom	C_6 (kJ mol^{-1} nm^6)	C_{12} (kJ mol^{-1} nm^{12})	Charge (e)
H14	8.464e-05	1.5129e-08	0.069
C6	0.0023406244	4.937284e-06	-0.308
H12	8.464e-05	1.5129e-08	0.069
H13	8.464e-05	1.5129e-08	0.069
C5	0.002340624	4.94E-06	0.219
H10	8.464e-05	1.5129e-08	-0.037
H11	8.464e-05	1.5129e-08	-0.037
C4	0.002340624	4.94E-06	-0.058
H8	8.464e-05	1.5129e-08	0.007
H9	8.464e-05	1.5129e-08	0.007
C3	0.002340624	4.94E-06	-0.058
H6	8.464e-05	1.5129e-08	0.007
H7	8.464e-05	1.5129e-08	0.007
C2	0.002340624	4.94E-06	0.219
H4	8.464e-05	1.5129e-08	-0.037

H5	8.464e-05	1.5129e-08	-0.037
C1	0.002340624	4.94E-06	-0.308
H1	8.464e-05	1.5129e-08	0.069
H2	8.464e-05	1.5129e-08	0.069
H3	8.464e-05	1.5129e-08	0.069
H_2O			
Atom	C_6 (kJ mol^{-1} nm^6)	C_{12} (kJ mol^{-1} nm^{12})	Charge (e)
OW	0.0026173456	2.634129e-06	-0.8476
HW1	0	0	0.4238
HW2	0	0	0.4238

S3. Molecular simulation process and pre-simulation optimization

S3.1 Molecular simulation process

The molecular simulations in this paper are shown in Fig. S1 below. Energy minimization involves eliminating the repulsive force within the system to prevent simulation crashes caused by excessive force on certain atoms at the start of the simulation. During the pre-equilibration, the system is designed to relax pressure and temperature from initial values to those near the expected values. This process also aims to eliminate any irregularities in the initial structure, ensuring that the overall simulation system reaches a state of balance and stability. During the formal simulation, an electric field is applied to the simulation system, initiating the collection of simulation data and statistical analysis.

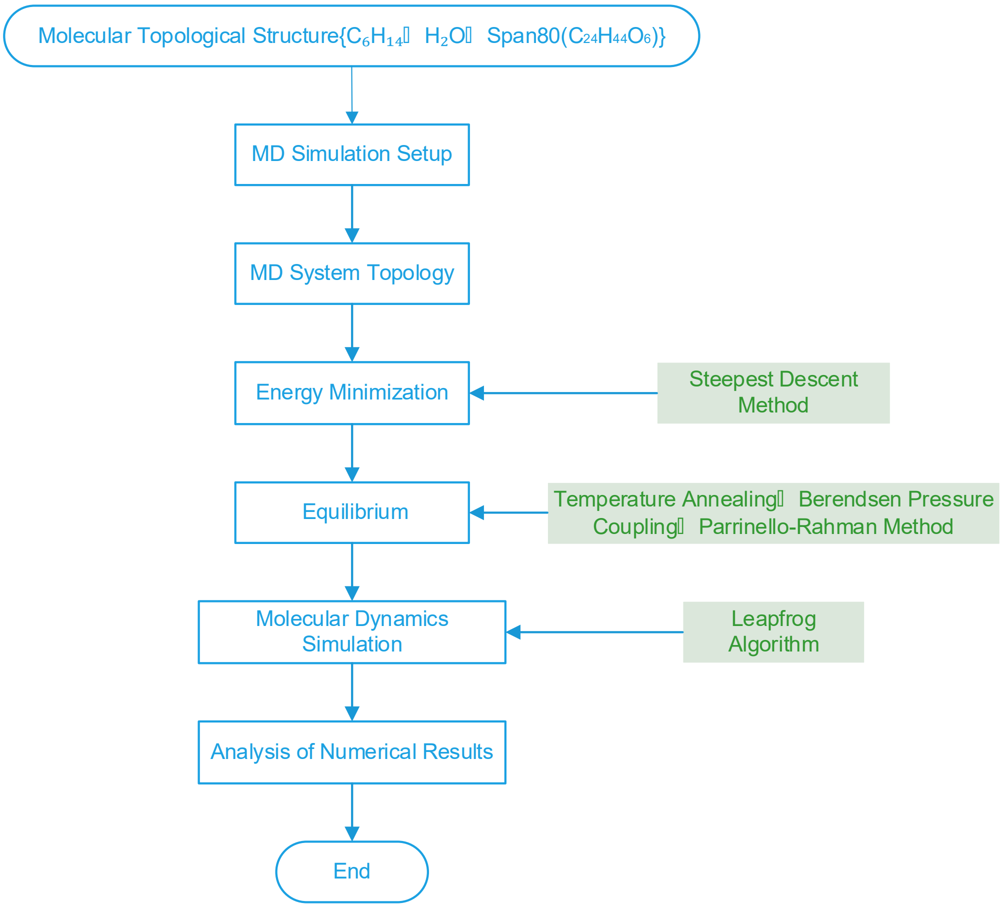

Fig. S1. Typical process of molecular dynamics simulation.

S3.2. Energy minimization

As shown in Fig. S2, the total potential energy at the end of the minimization process was observed to negative, which was expected for a system containing water molecules. From Table S2, the maximum force exerted in all simulated systems was below 200 kJ·mol^{-1}·nm^{-1}, indicating the complete convergence of the energy minimization.[4,5]

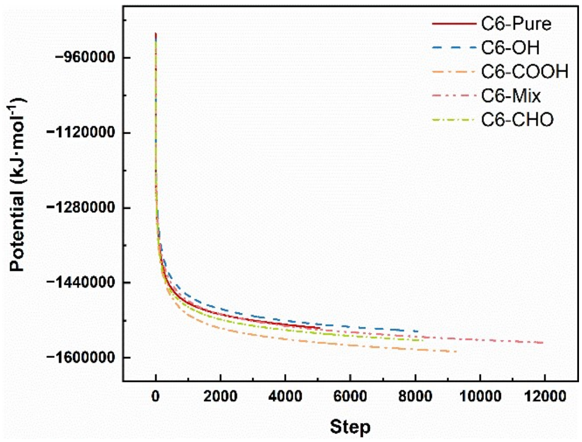

Fig. S2. Potential energy in the process of energy minimization.

Table S2 Data related to energy minimization of the system.

Types of model systems	Maximum force (F_{max}) [kJ·(mol·nm)$^{-1}$]	Steps converged to F_{max} < 200	Potential Energy
C_6-Pure	51.5	5055	-1536071.5
C_6-OH	175.4	8072	-1543019.1
C_6-COOH	187.5	9425	-1586955.4
C_6-CHO	101.1	8217	-1562544.1
C_6-Mix	198.5	11919	-1566992.4

S3.3. Equilibrium

As shown in Figures S3 and S4, the final temperature and pressure of the two systems basically fluctuated slightly around the preset value through the thermostat and barostat. Figure S5 showed that the total potential energy of the system was stable after equilibrium. So, it was suggested that the balanced structure could be used for formal simulations.[4,5]

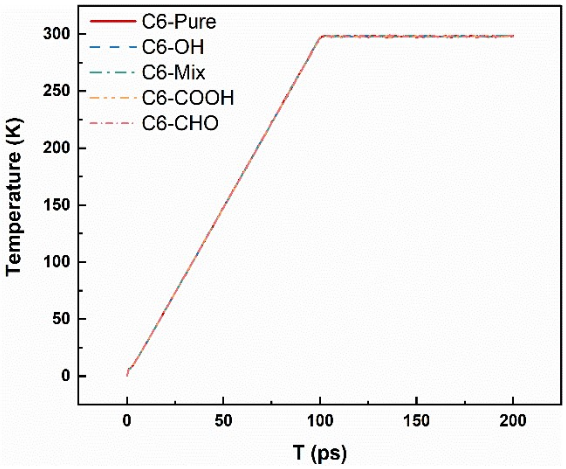

Figure. S3. Temperature during equilibrium.

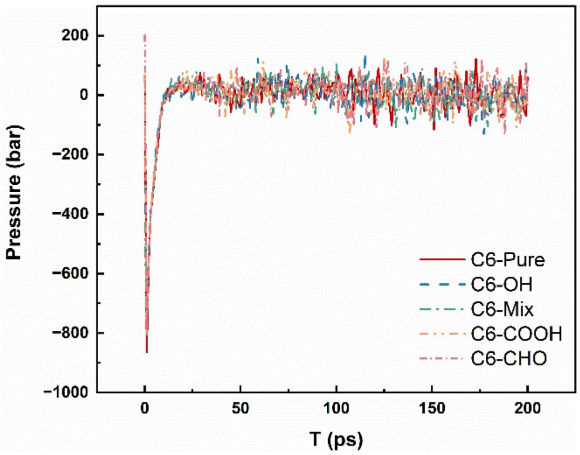

Figure. S4. Pressure during equilibrium.

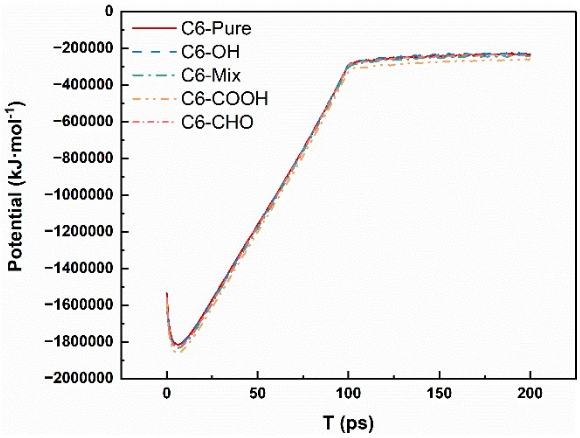

Figure. S5. Potential energy during equilibrium.

S4. Solvent accessible surface area (SASA)

The solvent molecule approaches the van der Waals surface of the central molecule, and the surface through which the center of the solvent molecule passes is referred to as the solvent accessible surface area (SASA), denoted in Figure S6.

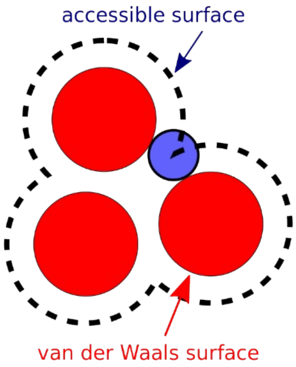

Figure. S6. Schematic diagram of solvent accessible surface area.

The formula for calculating the solvent accessible surface area is as follows.

$$SASA = 4\pi \sum_i r_i^2 \frac{m_{acc}(i)}{m} \qquad \text{(S3)}$$

where r_i is the sum of the van der Waals and solvent radii for each respective atom, $m_{acc}(i)$ is the number of dots on atom i not occluded by neighboring atoms and the summation is over all atoms in the molecule, and m is the number of points per sphere.

References

(1) C. Caleman and D. van der Spoel, Picosecond melting of ice by an infrared laser pulse: A simulation study, *Angew. Chem., Int. Ed.*, 2008, **47**, 1417-1420.

(2) M. Stroet, B. Caron, K. M. Visscher, D. P. Geerke, A. K. Malde and A. E. Mark, Automated Topology Builder Version 3.0: Prediction of Solvation Free Enthalpies in Water and Hexane, *J. Chem. Theory Comput.*, 2018, **14**, 5834-5845.

(3) N. Schmid, A. P. Eichenberger, A. Choutko, S. Riniker, M. Winger, A. E. Mark and W. F. van Gunsteren, Definition and testing of the GROMOS force-field versions 54A7 and 54B7, *Eur. J. Mech. B/Fluid*, **2011**, **40**, 843-856.

(4) P. S. Lee, R. T. Bradshaw, F. Marinelli, K. Kihn, A. Smith, P. L. Wintrode, D. J. Deredge, J. D. Faraldo-Gómez and L. R. Forrest, Interpreting Hydrogen-Deuterium Exchange Experiments with Molecular Simulations: Tutorials and Applications of the HDXer Ensemble Reweighting Software [Article v1.0], *Living J. Comp. Mol. Sci.*, 2018, DOI: 10.33011/livecoms.3.1.1521.

(5) M. J. Abraham, T. Murtola, R. Schulz, S. Páll, J. C. Smith, B. Hess and E. Lindahl,

GROMACS: High performance molecular simulations through multi-level parallelism from S10laptops to supercomputers, *SoftwareX*, 2015, **1**, 19-25.
